# Leveraging single-cell transcriptomic data to uncover immune suppressive cancer cell subsets in triple-negative canine breast cancers

**DOI:** 10.3389/fvets.2024.1434617

**Published:** 2024-09-23

**Authors:** Myung-Chul Kim, Nicholas Borcherding, Woo-Jin Song, Ryan Kolb, Weizhou Zhang

**Affiliations:** ^1^Veterinary Laboratory Medicine, Clinical Pathology, College of Veterinary Medicine, Jeju National University, Jeju, Republic of Korea; ^2^Research Institute of Veterinary Medicine, College of Veterinary Medicine, Jeju National University, Jeju, Republic of Korea; ^3^Department of Pathology and Immunology, Washington University School of Medicine in St. Louis, St. Louis, MO, United States; ^4^Laboratory of Veterinary Internal Medicine, College of Veterinary Medicine, Jeju National University, Jeju, Republic of Korea; ^5^Department of Pathology, Immunology and Laboratory Medicine, University of Florida College of Medicine, Gainesville, FL, United States; ^6^UF Health Cancer Center, University of Florida, Gainesville, FL, United States

**Keywords:** dog, immune checkpoint genes, interactome, scRNA-seq, triple-negative breast cancer

## Abstract

**Introduction:**

Single-cell RNA sequencing (scRNA-seq) has become an essential tool for uncovering the complexities of various physiological and immunopathological conditions in veterinary medicine. However, there is currently limited information on immune-suppressive cancer subsets in canine breast cancers. In this study, we aimed to identify and characterize immune-suppressive subsets of triple-negative canine breast cancer (TNBC) by utilizing integrated scRNA-seq data from published datasets.

**Methods:**

Published scRNA-seq datasets, including data from six groups of 30 dogs, were subjected to integrated bioinformatic analysis.

**Results:**

Immune modulatory TNBC subsets were identified through functional enrichment analysis using immune-suppressive gene sets, including those associated with anti-inflammatory and M2-like macrophages. Key immune-suppressive signaling, such as viral infection, angiogenesis, and leukocyte chemotaxis, was found to play a role in enabling TNBC to evade immune surveillance. In addition, interactome analysis revealed significant interactions between distinct subsets of cancer cells and effector T cells, suggesting potential T-cell suppression.

**Discussion:**

The present study demonstrates a versatile and scalable approach to integrating and analyzing scRNA-seq data, which successfully identified immune-modulatory subsets of canine TNBC. It also revealed potential mechanisms through which TNBC promotes immune evasion in dogs. These findings are crucial for advancing the understanding of the immune pathogenesis of canine TNBC and may aid in the development of new immune-based therapeutic strategies.

## Introduction

Immune checkpoint inhibitors (ICIs)-based immunotherapy has revolutionized cancer therapy, significantly altering the therapeutic landscape for many human cancers (1). Recent clinical trials in dogs have demonstrated promising results, with complete remission rates of 7.7% (2) and 9.5% (3, 4) in cases of advanced oral malignant melanoma following the administration of caninized anti-PD1 and anti-PD-L1 antibodies. In addition, anti-human CCR4 antibodies targeting tumor-infiltrating regulatory T cells elicited partial remission rates of 30% (5) and 71% (6) in canine models of prostate and bladder cancer, respectively. Despite a low overall response rate, dogs with non-melanoma solid tumors appear to clinically benefit from the treatment of anti-PD1 antibodies (7–9). ICIs in dogs have shown durability, although there has been one report of a fatality related to adverse events (4). Multiple mechanisms are assumed to be involved in dogs’ immune suppressive tumor microenvironment, including infiltration of immune-suppressive cells (10, 11) and expression of immune checkpoints that suppress T cell activity (2, 3, 12). Different types of immune modulatory therapies applied to dogs have shown that the tumor immune microenvironment (TiME) is the critical rate-limiting step in the cancer-immune cycle, influencing immune evasion and the effectiveness of cancer immunotherapy (2, 3, 13, 14). However, little information is available on the composition of the tumor microenvironment, its interaction with the tumor in the context of its microenvironment, and the specific tumor antigens in dogs (15).

Mammary gland tumors (MGT) are the most frequently diagnosed tumors in female dogs. Among the subtypes of MGT, triple-negative breast cancer (TNBC) is characterized by the absence of human epidermal growth factor receptor 2 (HER2), estrogen receptor (ER), and progesterone receptor (PR), with or without the basal-like type (cytokeratin 5/6-positive) (16). In dogs, the TNBC subtype is relatively common, accounting for approximately 27.9% of all canine MGT cases, although this proportion varies due to differences in inclusion criteria and methodology (16–20). Similar to humans, canine TNBC is more biologically aggressive than other MGT subtypes, resulting in poor prognosis (16, 17). The molecular mechanisms underlying the increased aggressiveness of canine TNBC remain largely unknown, but it is suggested that the epithelial-to-mesenchymal transition may play a role (17, 21).

In dogs, MGT has been suggested to induce immune suppression (10, 22, 23). Emerging studies have demonstrated the prognostic value of tumor-infiltrating leukocytes (TIL) in dogs with MGT (24). TNBC has been demonstrated to shape the suppressive TiME in humans (25, 26). In dogs with TNBC, tumor-infiltrating CD4^+^ T cells and tumor-associated macrophages have been negatively correlated with clinical outcomes (16, 27). Surgery is considered the primary treatment for canine MGT (28, 29). ICIs are the most studied forms of immunotherapy for TNBC (25, 30). New treatment strategies have been developed to reduce the incidence of local tumor recurrence and delay the metastatic progression of TNBC in dogs (31). TNBCs are biologically heterogeneous with low response to ICIs (30, 32) and represent a promising model system for comparative immunotherapy research in both canines and humans (16).

scRNA-seq has only recently begun to unlock the secrets of veterinary diseases, with applications to canine cells derived from osteosarcomas, chronic inflammatory enteropathy, Peyer’s patch, and hippocampus (33–37). In the present study, using published scRNA-seq datasets, we leveraged data integration to attest to the hypothesis that an immune modulatory subset of TNBCs is responsible for immune suppression via the interaction with effector T cells. As the first step toward cancer immunotherapy, the present study defined the immune suppressive subsets of canine TNBC at a high-resolution single-cell level and characterized the crosstalk between cancer cells and effector CD4^+^ and CD8^+^ T cells. The data presented in this study indicate potential mechanisms through which TNBCs shape the immune suppressive TiME, which was not addressed in the original publications (21, 27, 38). The integrated scRNA-seq analysis presented in this study will lay the groundwork for the development of methodologies to study cell subsets, their functions, and the complex cell–cell interactions in the TiME of different cancer types and other immune-related syndromes in veterinary medicine, including in dogs.

## Methods

### Single-cell RNA sequencing datasets

Canine scRNA-seq datasets and published studies are available in the Gene Expression Omnibus (GEO) database. These included a total of 30 scRNA-seq datasets: 10 datasets of peripheral blood mononuclear cells (PBMC) from five dogs with and five dogs without atopic dermatitis (GSE144730) (39), three datasets of peripheral blood T-cell receptor (TCR) αβ T cells (PBT) from three healthy dogs (GSE218355) (40), TNBC with or without *in vitro* vaccinia virus infection derived from two dogs (GSE142184) (38), a dataset of nuclei from lung tissue from a healthy dog (GSE183300) (41), four datasets of immune cells from bronchoalveolar lavage (BAL) (E-MTAB-9265) from four dogs (42), and eight datasets of immune cells from BAL from three dogs with and five dogs without idiopathic pulmonary fibrosis (IPF) (E-MTAB-9623) (43). Detailed sampling information was provided in the individual studies. Circulating leukocytes were isolated from canine blood using density gradient centrifugation (39, 40). BAL samples were isolated by instilling sterile saline solution into the airways through a bronchoscope channel, followed by fluid aspiration (42, 43). TNBC was confirmed by the lack of immunohistochemical expression of ER, PR, and HER2 (38). All datasets were integrated and subject to bioinformatic analysis. Each dataset was confirmed to use official canine gene symbols associated with human homologs. All studies used CanFam3.1 (*Canis lupus familiaris* genome assembly) for aligning reads to the reference genome.

### Single-cell RNA sequencing data integration and analysis

Seurat objects from all 30 samples were merged and integrated into a single object. R toolkit Seurat (v. 4.3.0) was used for the data processing, generating the Seurat object as an input file on RStudio (v. 4.2) for subsequent bioinformatic processes (44). Briefly, low-quality cells with either unique feature counts of less than 200 or over 5,000 or mitochondrial counts of more than 10% were filtered out. Samples were normalized using default settings. Preparation for integration used 3,000 anchor features. Principal component analysis (PCA) was used for linear dimensional reduction. Principal components 1 through 30 were utilized for further dimensional reduction, which was based on the most significant principal component (*p* < 1E-5) from the Jackstraw substitution test algorithm and the ranking of principal components based on the percentage of variance. Moreover, t-distributed stochastic neighbor embedding (t-SNE) was used for graph-based clustering with a resolution of 2.7.

The scDblFinder (v. 1.4.0) R package was used to remove potential doublets (45). Doublet prediction was conducted on each study group to account for batch effects. Following singlet selection, single-cell clusters were identified and labeled based on several criteria: markers from the original studies from which the dataset was sourced, canonical lineage markers, markers for rare and unique populations from previous publications, or unbiased cell type recognition using SingleR (v. 1.8.1) (46). The celldex package (v. 1.6.0) was used to utilize reference signatures of pure cell types to infer the cell of origin for each single cell (46).

### Differential gene expression analysis

A likelihood-ratio test was used to identify differential expression for individual clusters compared to all other cells. To identify cluster markers, the “*FindAllMarkers*” function was used in the Seurat package with the absolute log_2_-fold change threshold >0.25 and minimum percentage of cells where the gene is detected in a specific cluster >25%. To identify cluster differentially expressed genes (DEGs) for all clusters across groups, the “*FindMarkers*” function was used with the absolute log_2_-fold change threshold >0.36 and *p-*value <0.05.

### Gene set enrichment analysis and gene ontology analysis

Single-cell gene set enrichment analysis (GSEA) was performed using the escape R package (v. 1.6.0) (47). Gene sets were sourced from the Hallmark library of the Molecular Signature Database (48). Canine gene sets associated with cancer types were derived from previous publications (49–52). DEGs were also subjected to either Gene Ontology (GO) enrichment analysis using PANTHER annotation datasets with a species of *Canis lupus familiaris*^1^ or ShinyGO (v. 0.77) (53), a graphical gene-set enrichment tool with a species of dog. GO and Kyoto Encyclopedia of Genes and Genomes (KEGG) results were filtered with a *p*-value of <0.05 and a false discovery rate (FDR) of <0.05. The DittoSeq (v. 1.4.4) and pheatmap (v. 1.0.12) R packages were used to visualize gene sets that characterize specific molecular and biological pathways (54).

### Cell cycle analysis

Cell cycle assignment was performed by using the “*CellCycleScoring*” function and calling “*cc.genes.updated*.*2019*” in Seurat (44).

### Cell-to-cell interaction analysis

The CellChat R package (v. 1.4.0) was used to quantitatively infer intercellular communication networks from scRNA-seq data (55). Single cells derived from PBMC, αβ T, and TNBC groups were subject to interactome analysis.

The interaction analysis did not include PTPRC-non-immune cells, potential doublets, and clusters that were simultaneously assigned to both TNBC and immune cell groups. To find potential ligand-receptor pairs, the “*netVisual_bubble*” function was used with a threshold of *p-*value <0.01, as previously described (56).

### Statistical methods

Default statistical methods available within the Seurat package were used in this study, as previously described (56). A non-parametric Wilcoxon rank-sum test was used to compare the significance of two-sample differential expression in the “*FindAllMarkers*” function. A two-tailed unpaired Student’s *t*-test available within the ggpubr R package (v. 0.4.0) was used for statistical tests for the distribution of genes on count-level mRNA data.

## Results

### Standard pre-processing and quality control of the integrated scRNA-seq data

**Figure 1:**
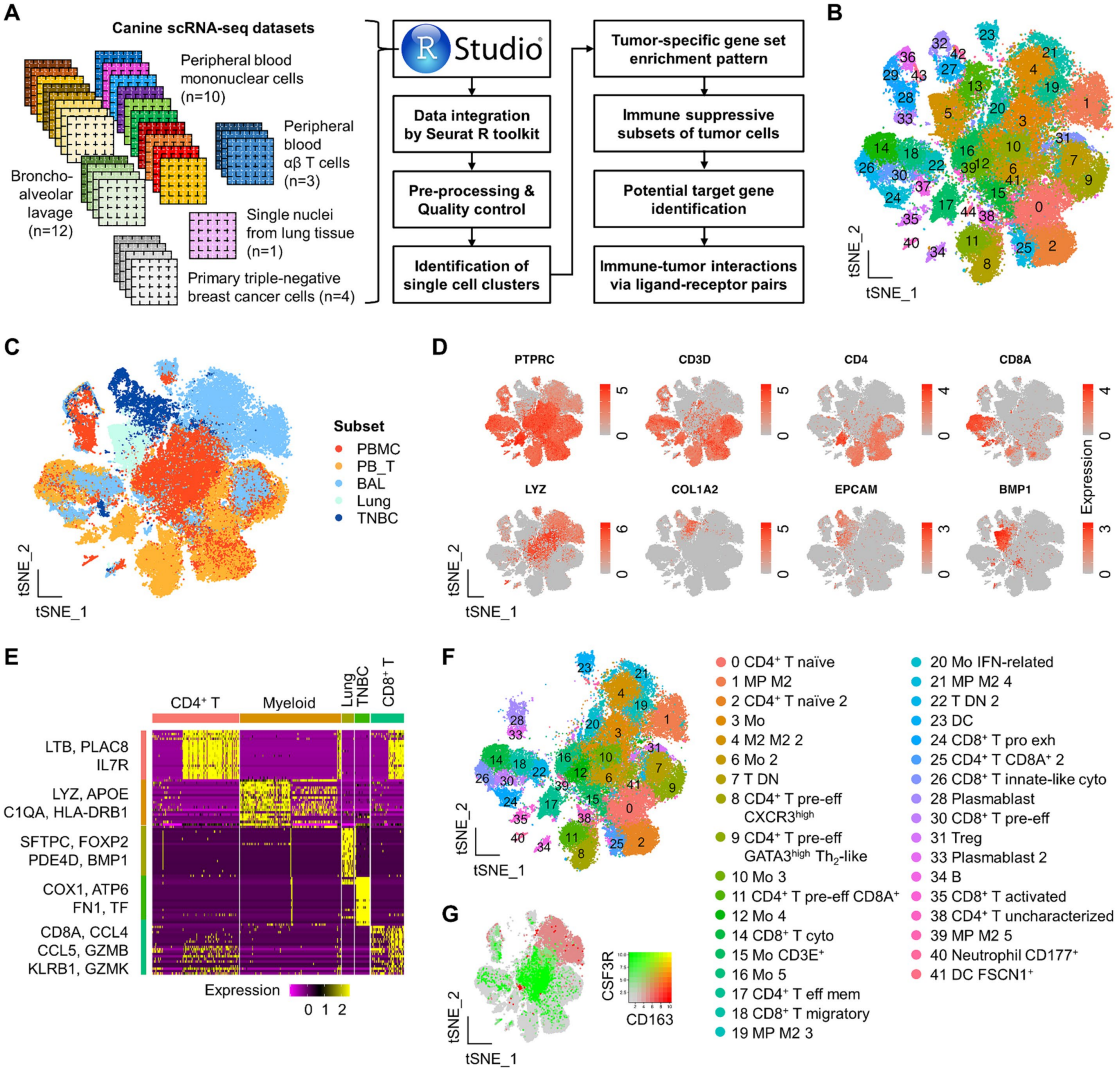
Study scheme and identification of major immune and TNBC clusters by integrated scRNA-seq analysis in dogs. **(A)** Study scheme. scRNA-seq datasets used in this study are present along with downstream analysis. **(B)** A total of 45 clusters identified from the integrated Seurat dataset are presented on the tSNE plot. **(C)** Single-cell tSNE distribution by the group. Overall, there is a distinct tSNE distribution of single cells across groups. **(D)** Canonical markers used to evaluate cell lineage are present on the tSNE plot. **(E)** Among the top 20 DEGs to define each major single-cell subset, representative genes are presented on the heatmap. **(F)** Identification of functionally distinct immune cells on the tSNE plot. **(G)** Simultaneous visualization of co-expression of CD163 and CSF3R on the tSNE plot. PBMC, peripheral blood mononuclear cells; PBT, peripheral blood TCR αβ T cells; BAL, bronchoalveolar lavage; Mo, monocytes; MP, macrophages; M1, M1-polarized; M2, M2-polarized; DC, dendritic cells; pre-eff, pre-effector; Eff, effector; Mem, memory; act, activated; Pro exh, progenitor exhausted; Treg, regulatory T cells; Cyto, cytotoxic.

The study workflow is presented in Figure 1A. The rationale for including these datasets is as follows: Immune cells derived from BAL samples were included as a control dataset (42, 43), given that macrophages were identified to be immune suppressive. Lung cells were included as a control dataset (41), given that airway epithelial cells were known to modulate innate mucosal immunity (57). PBMC derived from atopic dermatitis was included as a control dataset, given that immune cells were identified to be proinflammatory (39). A very low passage of primary TNBC cells that were confirmed to be not immortalized was included to attest to the hypothesis that dogs have immune modulatory subsets of TNBC (38). A summary of subject characteristics is presented in Supplementary Table S1. The standard pre-processing and rigorous quality control of scRNA-seq data integrated by studies are available (Supplementary Figure S1). The number of genes, percentage of reads that map to the mitochondrial genome, and percentage of canine ensemble genes detected in each study are shown (Supplementary Figure S1A). Mitochondrial genes were not identified in BAL and lung groups (Supplementary Figure S1B). The top 10 highest variable features among 3,000 features that exhibit high cell-to-cell variation in the integrated scRNA-seq dataset are shown (Supplementary Figure S1B). Principal components of 20 showing strong enrichment of features with low *p-*values were selected based on the JackStraw (Supplementary Figure S1C) and Elbow (Supplementary Figure S1D) plots. Following potential doublet exclusion (Supplementary Figure S1E), 69,035 single cells were obtained from PBMC (GSE144730, *n* = 20,078), peripheral blood TCR αβ T cells (GSE218355, *n* = 19,796), lung (GSE183300, *n* = 3,694), BAL (E-MTAB-9265, *n* = 4,240), BAL (E-MTAB-9623, *n* = 16,171), and TNBC (GSE142184, *n* = 5,056) (Supplementary Figure 1F).

### Identification of the major single-cell clusters by scRNA-seq

Following scRNA-seq data integration, a total of 45 clusters were identified in the master Seurat object (Figure 1B). We assessed the clustering performance by identifying the major cell types and found a clear separation of CD45^+^ (or *PTPRC*^+^) leukocytes, CD4^+^ T, CD8^+^ T (or *CD8A*^+^), myeloid (*LYZ*^+^), epithelial cells (*EPCAM*^+^ and/or *COL1A2*^+^), and lung (*BMP1*^+^) populations (Figures 1C,D). Out of the top 20 DEGs to define the major immune and non-immune subsets, representative genes are presented on the heatmap (Figure 1E). Functionally unique immune subsets from PBMC, αβ T, and BAL groups were further defined by various canonical markers and genes derived from previous studies (39, 40, 42, 43) (Figure 1F and Supplementary Figure S2). Unbiased cell type recognition was also used to define distinct clusters, supporting the clustering performance (Supplementary Figure S3).

In the master Seurat object, a total of 16 clusters of CD3^+^ T cells were identified, which mainly consisted of single-positive CD4^+^ or CD8^+^ cells, as well as a few double-positive (clusters 11 and 25) and double-negative (clusters 7 and 22, largely derived from BAL groups) subpopulations (Supplementary Figure S2A). CD4^+^ T cells were further classified into five transcriptionally unique subpopulations: *LEF1*^+^*SELL*^+^*CCR7*^+^ naïve (clusters 0 and 2), *CXCR3*^high^ pre-effector (cluster 8), *GATA3*^+^ Th2-like pre-effector (cluster 9), *CCR4*^high^*CXCR4*^high^ effector memory (cluster 17), *FOXP3*^+^ regulatory T cells (Treg) (cluster 31), and an uncharacterized population (cluster 38). Pre-effector CD4^+^ T cells were defined by a gradual increase in *HOPX* expression, a marker indicating pre-effector T cells poised for subsequent effector differentiation. A total of five CD8^+^ T clusters were identified in the master Seurat object. Clusters 14 and 26 showed high expression of genes related to killer cell lectin-like receptors (e.g., *KLRG1* and *KLRB1*) and cytotoxicity markers (e.g., *GZMA*, *PRF1*, and *NCR3*), suggesting these clusters represent cytotoxic CD8^+^ T cells (Supplementary Figure S2A). In addition, cluster 26 uniquely expressed *FCER1G*, indicating an innate-like phenotype. Cluster 24 displayed features of an exhausted progenitor phenotype, characterized by the expression of *CD7*, *TOX*, and, to some extent, *TCF7*, but lacked *CD27* expression. Cluster 35 showed high expression of genes associated with T cell activation, such as *CD69*, *JUND*, and KLF6, suggesting that these were activated CD8^+^ T cells. Cluster 30 expressed *HOPX* but showed lower levels of cytotoxicity-related genes, indicating it may represent a pre-effector type. Cluster 18 was marked by high expression of *CCL4*, *CCL5*, and *S100A4*, suggesting a migratory phenotype. Additionally, a small number of immune cells, such as gamma delta T cells and natural killer (NK) cells, were identified through unbiased cell type annotation but were not assigned to a single independent cluster (Supplementary Figure S3). Myeloid cells, defined by *LYZ* expression (Supplementary Figure S2B), were clearly separated on the tSNE plot based on the expression of *CSF3R* and *CD163*, which are classic markers for M1 and M2 macrophage polarization markers, respectively (Figure 1G). Myeloid clusters expressing *CSF3R* were identified as *ITGB2*^high^ monocytes (clusters 3, 6, 10, 12, 16, and 20) derived from peripheral blood (Supplementary Figure S3B). These clusters rarely expressed *CXCR2*, a granulocytic lineage marker (Supplementary Figure S2A).

Myeloid clusters expressing *CD163* were mainly *CD68*^high^ macrophages (clusters 1, 4, 19, 21, and 39) derived from BAL (bronchoalveolar lavage). Cluster 20 was further characterized as IFN-related monocytes due to the high expression of genes associated with interferon signaling pathways, such as *ISG15*, *MX1*, and *MX2*. We also identified *CD83*^+^*CD86*^+^*ITGAX*^+^ dendritic cells (DC) (cluster 23), *FSCN1*^+^ DC (cluster 41), and *CD177*^+^ neutrophils (cluster 40).

B cells (cluster 34) and plasmablasts (clusters 28 and 33) specifically expressed *MS4A1* and *IRF4*, respectively (Supplementary Figures S2A, S3A,B). Single cells derived from the lung were not immune cells but showed high expression of *BMP1* genes (Figure 1D and Supplementary Figure S2C). In the TNBC group, single cells exhibited specific expression of epithelial cell markers, such as *COL1A2*, *KRT14*, *CA2*, and *SPP1* (Supplementary Figure S2C). Notably, some immune and TNBC cells were assigned to the same clusters (29, 36, and 43), which is likely due to a similar global structure of RNA expression across single cells. Taken together, this integrated analysis successfully identified major distinct subsets of immune and TNBC cells at the single-cell level in dogs.

### Subsets of cancer cells have a distinct immune-suppressive phenotype

**Figure 2:**
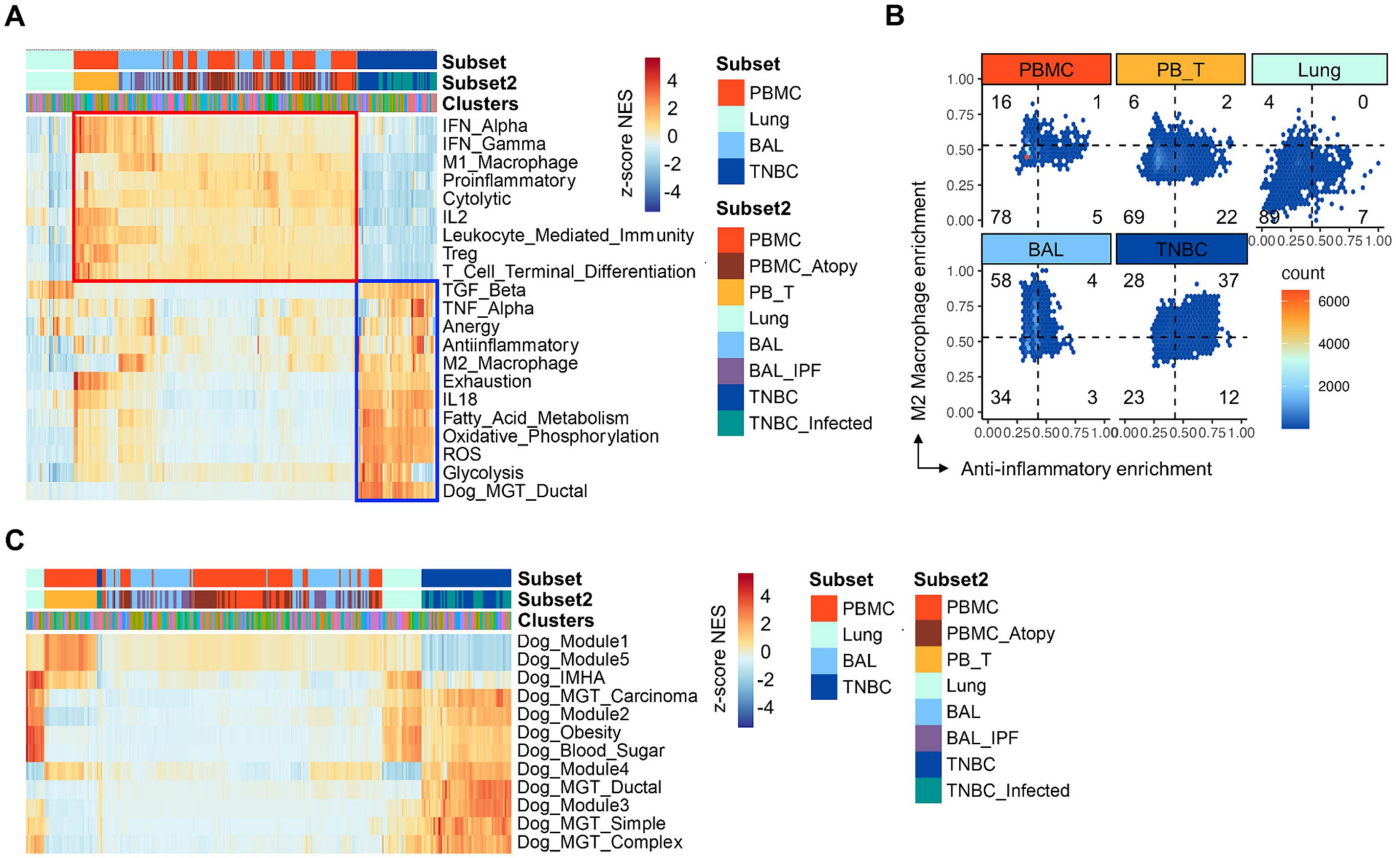
Gene set enrichment analysis of gene signatures associated with immune-related pathways. **(A)** GSEA. Immune-related pathways are present across subsets and clusters. TNBC clusters show marked enrichment with gene sets associated with metabolism, cytokine, and immune suppression, particularly including anti-inflammatory, M2 macrophage, and anergy signatures (blue boxed). Note that the specific enrichment of the canine gene set is associated with mammary gland tumors in TNBC. Contrary to TNBC, PBMC, and BAL show a general increase in the enrichment with gene sets associated with inflammatory responses, such as interferons, M1 macrophages, and proinflammatory (red boxed). **(B)** The hex density enrichment plot reveals the enrichment pattern of the indicated immune-related pathways across groups. The TNBC group exhibits a more anti-inflammatory phenotype than other groups, as evidenced by the TNBC-preferential shift toward the anti-inflammatory and M2 macrophage signatures. Note intrinsic, significant enrichment of M2 macrophage signature in single cells from BAL group. **(C)** GSEA. Enrichment of canine gene signatures is present across subsets and clusters. GSEA, gene set enrichment analysis; PBMC; peripheral blood mononuclear cells; PB_T, peripheral blood TCR αβ T cells; TNBC, triple-negative breast cancer; BAL, bronchoalveolar lavage; TNBC_infected, TNBC subset infected with oncolytic vaccinia virus; Dog_Module 1, gene set defining canine pulmonary carcinoma; Dog_Module 2, gene set associated with canine malignant melanoma; Dog_Module 3, gene set associated with canine osteosarcoma; Dog_Module 4, gene set associated with canine B cell lymphoma; Dog_Module 5, gene set associated with canine T cell lymphoma; Dog_IMHA, gene signature associated with canine immune-mediated hemolytic anemia.

Clinical trials have revealed the presence of an immune suppressive TiME in various types of canine cancers (5). However, whether canine TNBCs possess an immune-suppressive TiME remains unknown. To investigate this, we performed GSEA using various gene sets associated with immune-associated pathways (Figure 2). Overall, we identified two distinct patterns of gene set enrichment. First, PBMC were preferentially enriched with inflammatory gene sets, such as interferon signaling, M1 macrophage, proinflammatory, and leukocyte-mediated immunity (Figure 2A, red boxed). Specifically, αβ T cells within the PBMCs showed significant enrichment of T cell-specific gene signatures, including those associated with Treg and terminal T cell differentiation. Second, TNBCs exhibited preferential enrichment of immune suppressive gene sets, such as those related to TGF-β, TNF-α, anergy, anti-inflammatory responses, M2 macrophages, and T cell exhaustion (Figure 2A, blue boxed). Notably, the anti-inflammatory and M2 macrophage enrichment patterns were particularly specific to TNBC compared to other groups (Figure 2B). Additionally, TNBCs were enriched with gene sets associated with alternative metabolic pathways and oxidative stress (Figure 2A). Importantly, canine-specific gene sets were also enriched (Figure 2C), demonstrating the reliability of the GSEA results in this study. Consistent with previous scRNA-seq findings in dogs (43), BAL samples affected by IPF showed single-cell clusters that were highly enriched with the M2 macrophage gene set (Figures 2A,B). Moreover, lung cells showed only sporadic enrichment patterns of several gene sets, such as TGF-β and anergy (Figures 2A,B). There was no noticeable difference in the enrichment pattern between healthy and atopic dermatitis conditions of PBMC. Taken together, GSEA confirmed that TNBC contributes to an immune-suppressive TiME in dogs, providing a rationale for further analysis with a focus on identifying specific TBNC clusters that might have led to the distinct immune-suppressive phenotype.

### Identification and characterization of cancer cell subsets within TNBCs

**Figure 3:**
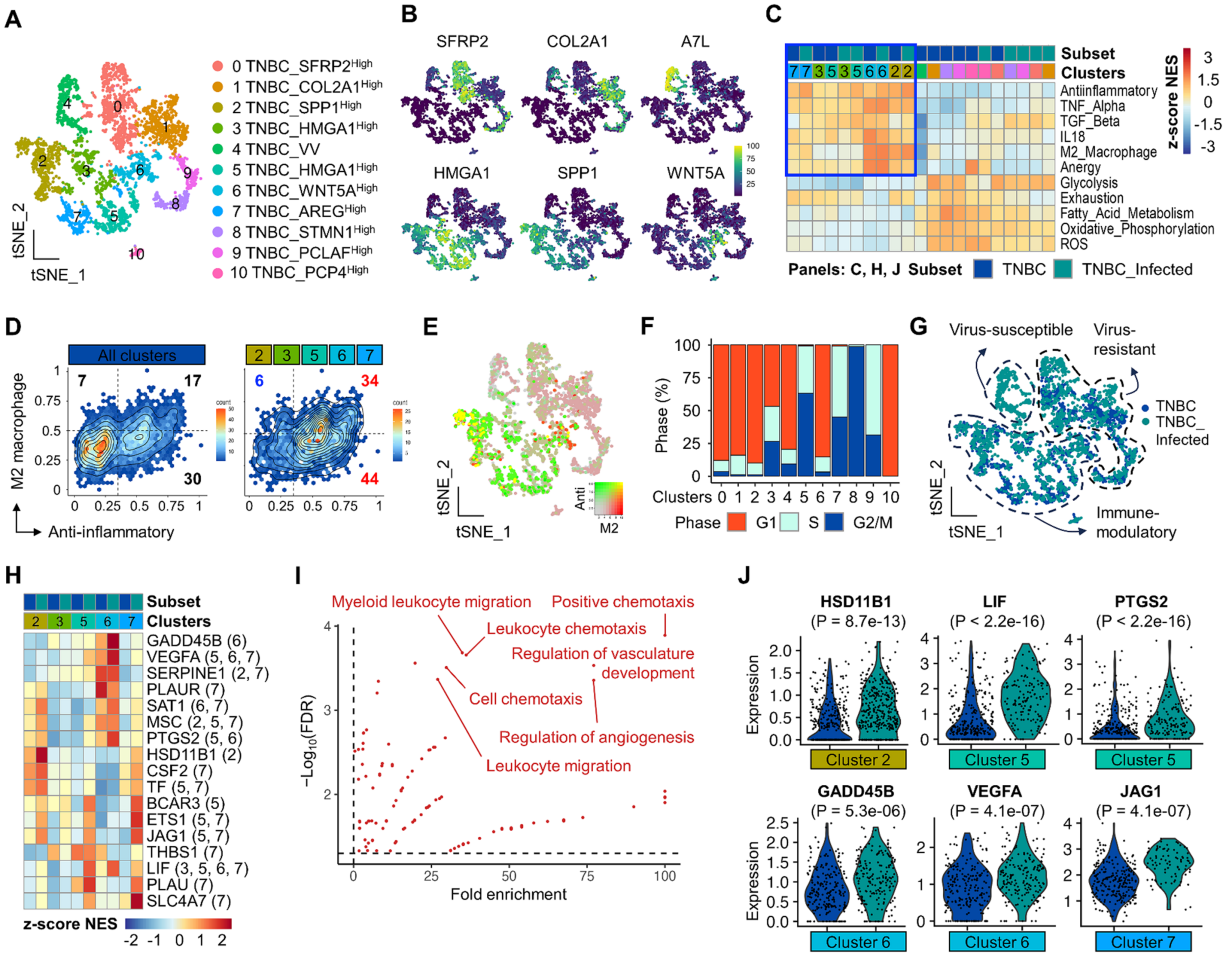
Identification and characterization of immune suppressive TNBC subsets and potential candidates by scRNA-seq. **(A)** A total of 11 major TNBC clusters are presented on the tSNE plot. **(B)** Representative markers to define clusters are present on the feature plot. **(C)** GSEA shows preferential enrichment patterns for gene sets associated with M2 macrophage, TGF-β, IL18, anergy, anti-inflammatory, and TNF-α in clusters 2, 3, 5, 6, and 7 TNBC clusters. **(D)** Hex density enrichment plot reveals that indicated TNBC subsets are more anti-inflammatory compared to the entire population of TNBC. Red and blue numbers on each quadrant illustrate upward and downward trends, respectively, compared to control. **(E)** Simultaneous visualization of co-enrichment of anti-inflammatory and M2 macrophage signatures on the tSNE plot. **(F)** Cell cycle profile across TNBC subsets. **(G)** Molecular classification of TNBC clusters according to viral infection, transcriptional features, and functional enrichment. **(H)** Differentially upregulated genes that belong to gene sets highlighted in **(C)** are present across TNBC clusters. Numbers in the parenthesis indicate the origin of statistically significant DEGs in TNBC clusters. **(I)** Gene ontology analysis using DEGs affected by viral infection. Highly enriched and statistically significant representative GO terms are shown, which are associated with the regulation of angiogenesis and leukocyte chemotaxis. **(J)** The violin plot reveals differential expression of indicated genes in TNBC subsets. A comparison between the two groups of interest was made using a two-tailed unpaired Student’s *t*-test.

To scrutinize functionally unique cancer cell subpopulations, we performed sub-clustering of all cancer cells and defined 11 sub-clusters (Figure 3A). Each cluster was separated in the tSNE plot, demonstrating prominent intratumoral heterogeneity and distinct global structure of transcriptomes across clusters (Supplementary Figure S4). Representative markers for each TNBC cluster are available (Figure 3B, Supplementary Figures S4A,B, and Supplementary Table S2). For example, the expression of genes associated with immune suppression, such as *SPP1*, *HMGA1* and *WNT5A*, was mainly identified in clusters 2, 3, 5, 6, and 7. Clusters 0 and 1 were characterized by specific expressions of *SFRP2* and *COL2A1*, respectively.

Subsequently, we performed functional enrichment analysis to investigate cancer cell subsets that could contribute to immune suppression. Interestingly, GSEA identified clusters 2, 3, 5, 6, and 7 that were preferentially enriched with gene sets associated with immune suppression, such as anti-inflammatory, M2 macrophage, anergy, IL-18, TNF-*α*, and TGF-β (Figure 3C, blue boxed, Figure 3D, and Supplementary Figure S4C). The enrichment with an anti-inflammatory signature tended to increase in virus-infected clusters relative to non-viral-infected cancer cell clusters (Figure 3D). Among them, clusters 2 and 6 showed simultaneous enrichment of anti-inflammatory and M2 macrophage gene sets, indicative of potential immune-suppressive cancer cells (Figure 3E). KEGG analysis using cluster markers revealed that cluster 2 was significantly associated with extracellular matrix-receptor interaction (Supplementary Figure S4D). Cluster 6 was significantly associated with blood vessel development and cell migration (Supplementary Figure S4D). These enrichment patterns were not associated with cell cycle alteration (Figure 3F), given that viral infection tended to arrest cell cycle progression, especially in clusters 2 and 6 (Supplementary Figure S4E). These GSEA and expression analyses classified cancer cells into virus-susceptible, virus-resistant, and immune-modulatory TNBC subsets (Figure 3G). During virus infection, bystander cancer cells can change behavior, potentially favoring immune evasion (58). The increasing tendency of anti-inflammatory enrichment in virus-infected, particularly immune-modulatory cancer cells, might imply genes that can play a key role in shaping an immune-suppressive TiME. Among genes belonging to the immune suppressive signatures, we identified 40 DEGs across cancer cell subsets related to viral infection status (Figure 3H, Supplementary Table S3, and Supplementary Figure S4F). Overall, these genes were significantly enriched with many biological processes, particularly angiogenesis and leukocyte chemotaxis (Figure 3I, Supplementary Figures S4F,G, and Supplementary Table S4). In support of this, *VEGFA* that belonged to these GO terms significantly increased in virus-infected cancer cell clusters compared to non-infected clusters (Figure 3J and Supplementary Figure S4G). Interestingly, *VEGFC*, which is associated with immune suppression during canine mammary cancer development (22), was also significantly upregulated in a subset of the cancer cell population (Supplementary Figure S4G). Other potential immune modulatory candidate genes, such as *HSD11B1*, *LIF*, *PTGS2*, *GADD45B*, and *JAG1*, were present (Figure 3J).

### Identification and characterization of cell-to-cell interaction between cancer cells and PBMCs

**Figure 4:**
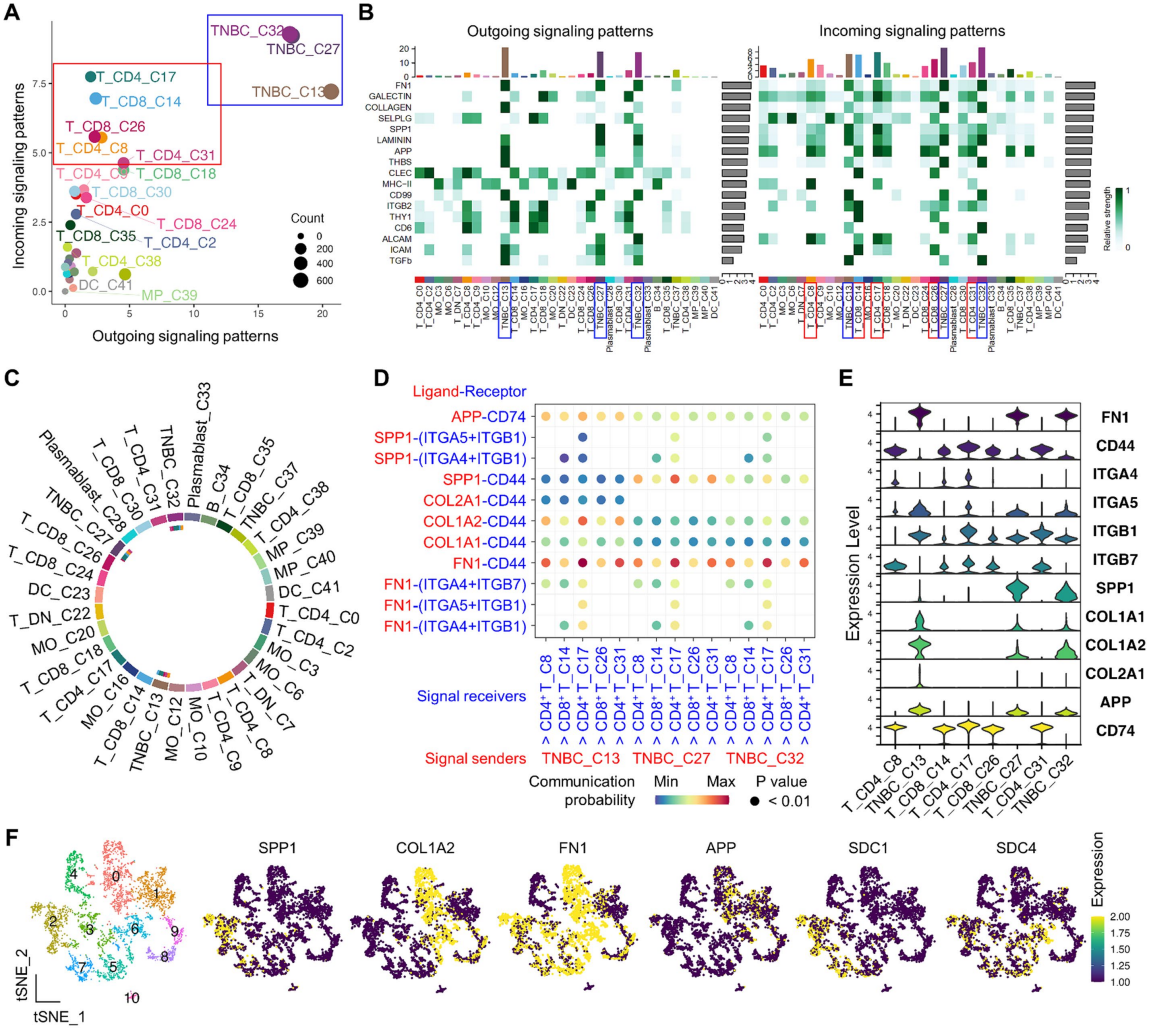
CellChat-based identification of immune-to-TNBC interactions by scRNA-seq. **(A)** Institutive visualization of the dominant signal senders (blue box) and receivers (red box) on the scatter plot. **(B)** Overall, outgoing and incoming signaling patterns of significant pathways across clusters are presented. Bars refer to the sum of the original computed interaction strength in each column and row. **(C)** Visualization of cell–cell communication from indicated TNBC to T clusters is present on the chord diagram. The inner thinner bar colors represent the targets that receive signals from the corresponding outer bar. **(D)** Identification of significant interactions and key ligand and receptor pairs from indicated TNBC to T clusters on the bubble plot. **(E)** Expression of representative ligand and receptor genes that belong to SPP1, FN1, APP, and collagen signaling pathways is present across clusters in the violin plot. **(F)** Identification of TNBC ligand gene expression on the functionally unique TNBC subpopulations from the sub-clustered group.

Tumor-immune cell interaction, a hallmark of cancer immunology, plays a critical role in T cell exhaustion within the TiME, leading to ineffective cancer immunotherapy. As a potential mechanism through which cancer cells induce the exhaustion stage of tumor-infiltrating T cells, we hypothesize that peripheral T cells have the potential to interact with cancer cells via distinct ligand-receptor pairs. Single-cell clusters from cancer cells and PBMC groups were subject to CellChat analysis to decipher coordinated tumor-immune interactions (Supplementary Figure S5A). While analyzing the interactome, we found two major patterns of cell–cell interaction. First, there were distinct subsets of cancer cells (clusters 13, 27, and 32) that strongly sent signals to other cells in a paracrine fashion or to themselves via an autocrine pathway (Figures 4A,B, blue boxed). Second, effector CD4^+^ (cluster 8 pre-effector *CXCR3*^high^, cluster 17 effector memory, and cluster 31 Treg) and CD8^+^ (cluster 14 cytotoxic and cluster 26 innate-like cytotoxic) T cells were able to receive signals (Figures 4A,B, red boxed, and Supplementary Figure S5B). Afterward, we focused on these clusters to identify significant signaling pathways and ligand/receptor pairs (Figure 4C).

After extensive interactome analysis, we identified key ligand and receptor pairs, which were mainly derived from secretory (SPP1), cell–cell intact (APP), and extracellular matrix-receptor (FN1 and collagen) pathways (Figure 4D and Supplementary Figure S5C). For example, the TNBC ligand (COL1A2, SPP1, and FN1) and receptors (SDC1, SDC4, ITGAV + ITGB1, ITGAV + ITGB5, and ITGA5 + ITGB1) showed high communication probability (Supplementary Figure S5D). Interestingly, T cell receptors CD44 and CD74 showed high communication probability, contributing most to the outgoing signaling of the representative ligands from cancer cells (Supplementary Figure S5E). The ligand and receptor genes showed high expression levels in the interacting cancer cells and T clusters (Figure 4E). Finally, the TNBC ligand and receptor genes (*SPP1*, *COL1A2*, *FN1*, *APP, SDC1*, and *SDC4*) were also expressed in immune-modulatory cancer cell subpopulations (clusters 2, 3, 5, 6, and 7) (Figure 4F), suggesting potential immune suppressive mechanisms mediated by cancer-T cell and cancer-cancer cell interaction.

## Discussion

We utilized open-source canine scRNA-seq datasets in the present study, including primary TNBC and peripheral immune cells. We investigated the mechanism through which TNBC induces immune suppression in dogs. The integrated scRNA-seq analysis in this study reveals immune suppressive canine TNBC subsets, which were identified to preferentially interact with TNBC subsets and effector types of CD4^+^ and CD8^+^ T cells. Potential mechanisms through which canine TNBC cells shape the immune suppressive TiME are suggested to regulate angiogenesis and immune cell infiltration.

By leveraging integrated scRNA-seq analysis, we identified transcriptionally distinct cancer cell subsets with potentially different cancer immune properties and interactions with T cells, which cannot be captured by bulk RNA-seq. Of note, while corroborating previous findings, e.g., viral-resistant DDIT4^+^ cancer cells (clusters 0, 1, 8, and 9) (38), bystander cancer cells identified by the previous study were reinterpreted to immune modulatory TNBC subsets in this study. The immune modulatory TNBC subsets identified in this study are functionally unique, as indicated by the expression of specific cluster markers, such as *HMGA1*, *SPP1*, and *WNT5A*. Interestingly, these genes are closely linked to the development and potential immune evasion mechanisms in both canine (59, 60) and human TNBC (61, 62). For example, *HMGA1*, a downstream gene of PD-L1 that regulates cancer immunity in human TNBC (63), was exclusively expressed in immune-modulatory TNBC subsets. *WNT5A*-induced Wnt signaling promotes immune escape in TNBC through multiple pathways, such as creating a hypoxic microenvironment, suppressing immune responses, and excluding T-cell infiltration (64). Similarly, SPP1, expressed on malignant cells, contributes to T-cell suppression via CD44-mediated binding (65). Therefore, we suggest that HMGA1^+^SPP1^+^ or WNT5A^+^ TNBC cells may play a role in immune evasion and could be potential targets for anticancer immunotherapy. The functional enrichment of immune-suppressive gene sets in these TNBC subpopulations further supports the conclusions of this study.

Immunosuppressive pathways could play a prominent role in the resistance of tumor cells to oncolytic viral infection (58). Accordingly, we explored oncolytic viral infection as an anti-inflammatory factor and identified candidate genes involved in immune evasion and mechanisms through which TNBC escapes immune surveillance by the host. GO analysis of DEGs in cells infected by the virus revealed two key signaling pathways: angiogenesis and leukocyte chemotaxis. Angiogenesis is a hallmark of canine mammary gland tumorigenesis, with VEGF signaling being critical in the pathophysiology of canine TNBC (66). In addition, angiogenesis is significantly correlated with immune suppression in dogs (22). In this study, immune-modulatory TNBC subsets affected by viral infection significantly upregulated *VEGFA* and *VEGFC*. Angiogenesis promotes the infiltration of various immune-suppressive cells into MGT in dogs (24). Indeed, VEGFC released by canine MGT contributes to immune suppression by recruiting Treg and myeloid-derived suppressor cells (MDSCs) (22). Moreover, the infiltration of Treg (67) and tumor-associated macrophages (TAMs) (68) is promoted by VEGF signaling in canine MGT. These recruited immune-suppressive cells have been strongly suggested to inhibit anticancer T-cell activity, contributing to poor prognoses in canine MGT and TNBC (22, 24, 27). Based on previous findings and our current results, we postulate that TNBC promotes cancer immunity through angiogenesis and VEGF-mediated immune cell infiltration. Future research is warranted to elucidate the mechanisms by which canine TNBC modulates cancer immunity through the regulation of other candidate genes. For example, in this study, TNBC cancer cells were inferred to interact specifically with each other, suggesting the presence of autocrine and paracrine communications. Based on this observation, we propose that the candidate genes may form a positive feedback loop, amplifying the anti-inflammatory signaling pathways within cancer cells. Indeed, oncolytic virus-regulated candidate genes, such as *PTGS2*, *GADD45B*, and *JAG1*, have been involved in autocrine and/or paracrine signaling within the TiME of human TNBC (69–71). Therefore, targeting therapeutic strategies to disrupt this potential intertumoral feedback loop may provide an effective approach to normalizing the TiME.

The interactome analysis reveals that cancer cells directly modulate T-cell activity, potentially promoting immune suppression. In this study, we suggest that *SPP1*^+^, *FN1*^+^, or *COL1A2*^+^ cancer cells may be a key subset responsible for T-cell suppression, where CD44 likely acts as an immune checkpoint in dogs. Indeed, the SPP1-CD44 interaction has been demonstrated to suppress infiltrating effector T-cell activity in various types of cancers (65, 72). This interaction may occur between proteins of both tumor and non-tumor origin (73). For instance, the binding of CD44^+^ tumor-infiltrating T cells to type I collagen has been demonstrated to induce a more aggressive phenotype in malignant melanoma (74). In addition, FN1^+^ TNBC cells are associated with increased CD8^+^ T cell infiltration and immune suppression (75). Although there is limited information on cell–cell interactions in canine TNBCs, numerous studies support the anti-inflammatory roles of *SPP1*, *FN1*, and *COL1A2* in canine MGT and TNBC (59, 76, 77). Investigating the impact of the TNBC-Treg interaction could provide further insights into immune evasion mechanisms. It might be associated with the direct induction of Treg, which supports CD4^+^ T cell-mediated poor prognosis of canine mammary carcinoma and TNBC. In the present study, interactions between TNBC subsets may contribute to the development of more aggressive TNBC phenotypes, such as increased invasiveness and metastatic potential. Notably, strong communication probabilities between the breast cancer ligand FN1 and syndecan receptors, such as SDC1 and SDC4, have been significantly involved in promoting a brain metastatic TiME (78). SDC1, a key immunity-related gene that is highly expressed in TNBC patients, facilitates immune escape by excluding tumor-infiltrating lymphocytes (79). Future studies are warranted to explore the clinical relevance of the binding of TNBC ligands to receptors on CD44^+^ T and other TNBC subsets in driving immune suppression in canine MGT.

This study leveraged scRNA-seq integration to define immune-suppressive subsets of TNBC at a high-resolution single-cell level and to characterize the crosstalk between cancer cells and effector CD4^+^ and CD8^+^ T cells. The results presented in this study will be valuable in demonstrating that canine TNBC shapes an immune-suppressive tumor microenvironment, which is mediated by interactions between immune cells and TNBC, and affected by exhausted CD44^+^ effector CD4^+^ T cells.

We acknowledge that this study has certain limitations. First, tumor-infiltrating immune cells were not analyzed, and peripheral immune cells from non-tumor-bearing dogs were used for the analysis. Although PBMCs may infiltrate tumor sites, interact with tumor cells, and become exhausted through immune-tumor interactions, future studies must confirm this in canine TNBCs. Second, we did not fully characterize the TiME in terms of the composition of immune components within canine TNBC. In addition, although the low passage of TNBC cells were analyzed, they may not fully represent the *in vivo* tumor microenvironment. Nevertheless, the results presented in this study will be valuable in identifying potential interaction among immune-modulatory TNBC subsets, which may contribute to the immunopathogenesis of TNBC in dogs. Third, despite the well-established integration methodology provided by Seurat, potential institutional or batch effects across scRNA-seq datasets might have occurred during the analysis. Finally, functionally validated canine gene sets were not utilized during GSEA. Although humans and dogs share a high degree of homology in corresponding sequences and orthologous genes, especially in well-conserved interspecies immunological functions (80), a more accurate assessment of immune-related functions should be performed using canine-specific gene sets.

Immunotherapy for dogs still lags far behind human treatment (15). Future scRNA-seq studies are warranted to map the immune landscape of canine MGT using paired samples of tumor cells, PBMC, and TILs identify novel and targetable immune checkpoint genes in tumor-bearing dogs.

## Data Availability

The original contributions presented in the study are included in the article/Supplementary material, further inquiries can be directed to the corresponding author.

## Footnotes

^1^ http://geneontology.org/

## Supplementary material

SUPPLEMENTARY TABLE S1 A summary of subject characteristics.

SUPPLEMENTARY TABLE S2 A list of cluster markers defining TNBC subpopulations.

SUPPLEMENTARY TABLE S3 A list of DEGs defining immune suppressive TNBC subsets and their enrichment gene sets.

SUPPLEMENTARY TABLE S4 A list of GO biological processes using DEGs that are listed in the Supplementary Table S3.

## References

[1] Waldman AD Fritz JM Lenardo MJ. A guide to cancer immunotherapy: from T cell basic science to clinical practice. Nat Rev Immunol. (2020) 20:651–68. doi: 10.1038/s41577-020-0306-5, PMID: 32433532 PMC7238960

[2] Maekawa N Konnai S Nishimura M Kagawa Y Takagi S Hosoya K. PD-L1 immunohistochemistry for canine cancers and clinical benefit of anti-PD-L1 antibody in dogs with pulmonary metastatic oral malignant melanoma. NPJ Precis Oncol. (2021) 5:10. doi: 10.1038/s41698-021-00147-6, PMID: 33580183 PMC7881100

[3] Igase M Inanaga S Tani K Nakaichi M Sakai Y Sakurai M. Long-term survival of dogs with stage 4 oral malignant melanoma treated with anti-canine PD-1 therapeutic antibody: a follow-up case report. Vet Comp Oncol. (2022) 20:901–5. doi: 10.1111/vco.12829, PMID: 35535636

[4] Igase M Nemoto Y Itamoto K Tani K Nakaichi M Sakurai M. A pilot clinical study of the therapeutic antibody against canine PD-1 for advanced spontaneous cancers in dogs. Sci Rep. (2020) 10:18311. doi: 10.1038/s41598-020-75533-4, PMID: 33110170 PMC7591904

[5] Maeda S Motegi T Iio A Kaji K Goto-Koshino Y Eto S. Anti-CCR4 treatment depletes regulatory T cells and leads to clinical activity in a canine model of advanced prostate cancer. J Immunother Cancer. (2022) 10:e003731. doi: 10.1136/jitc-2021-003731, PMID: 35131860 PMC8804701

[6] Maeda S Murakami K Inoue A Yonezawa T Matsuki N. CCR4 blockade depletes regulatory T cells and prolongs survival in a canine model of bladder cancer. Cancer Immunol Res. (2019) 7:1175–87. doi: 10.1158/2326-6066.CIR-18-0751, PMID: 31160277

[7] Igase M Inanaga S Nishibori S Itamoto K Sunahara H Nemoto Y. Proof-of-concept study of the caninized anti-canine programmed death 1 antibody in dogs with advanced non-oral malignant melanoma solid tumors. J Vet Sci. (2024) 25:e15. doi: 10.4142/jvs.23144, PMID: 38311328 PMC10839171

[8] Xu S Xie J Wang S Tang N Feng J Su Y. Reversing stage III oral adenocarcinoma in a dog treated with anti-canine PD-1 therapeutic antibody: a case report. Front Vet Sci. (2023) 10:1144869. doi: 10.3389/fvets.2023.1144869, PMID: 37252387 PMC10219605

[9] Deguchi T Maekawa N Konnai S Owaki R Hosoya K Morishita K. Enhanced systemic antitumour immunity by Hypofractionated radiotherapy and anti-PD-L1 therapy in dogs with pulmonary metastatic Oral malignant melanoma. Cancers. (2023) 15:13. doi: 10.3390/cancers15113013, PMID: 37296981 PMC10252299

[10] Kim JH Hur JH Lee SM Im KS Kim NH Sur JH. Correlation of Foxp3 positive regulatory T cells with prognostic factors in canine mammary carcinomas. Vet J. (2012) 193:222–7. doi: 10.1016/j.tvjl.2011.10.022, PMID: 22130461

[11] Gulay KCM Aoshima K Maekawa N Suzuki T Konnai S Kobayashi A. Hemangiosarcoma cells induce M2 polarization and PD-L1 expression in macrophages. Sci Rep. (2022) 12:2124. doi: 10.1038/s41598-022-06203-w, PMID: 35136176 PMC8826392

[12] Cascio MJ Whitley EM Sahay B Cortes-Hinojosa G Chang L-J Cowart J. Canine osteosarcoma checkpoint expression correlates with metastasis and T-cell infiltrate. Vet Immunol Immunopathol. (2021) 232:110169. doi: 10.1016/j.vetimm.2020.110169, PMID: 33387703

[13] Rebhun RB York D Cruz SM Judge SJ Razmara AM Farley LE. Inhaled recombinant human IL-15 in dogs with naturally occurring pulmonary metastases from osteosarcoma or melanoma: a phase 1 study of clinical activity and correlates of response. J Immunother Cancer. (2022) 10:493. doi: 10.1136/jitc-2022-004493, PMID: 35680383 PMC9174838

[14] Panjwani MK Atherton MJ MaloneyHuss MA Haran KP Xiong A Gupta M. Establishing a model system for evaluating CAR T cell therapy using dogs with spontaneous diffuse large B cell lymphoma. Onco Targets Ther. (2020) 9:1676615. doi: 10.1080/2162402X.2019.1676615 PMC6959441 32002286

[15] Klingemann H. Immunotherapy for dogs: still running behind humans. Front Immunol. (2021) 12:665784. doi: 10.3389/fimmu.2021.665784, PMID: 34421888 PMC8374065

[16] Abadie J Nguyen F Loussouarn D Peña L Gama A Rieder N. Canine invasive mammary carcinomas as models of human breast cancer. Part 2: immunophenotypes and prognostic significance. Breast Cancer Res Treat. (2018) 167:459–68. doi: 10.1007/s10549-017-4542-8, PMID: 29063312 PMC5790838

[17] Varallo GR Gelaleti GB Maschio‑Signorini L Moschetta MG Lopes JR de Nardi A. Prognostic phenotypic classification for canine mammary tumors. Oncol Lett. (2019) 18:6545–53. doi: 10.3892/ol.2019.11052, PMID: 31807173 PMC6876320

[18] Gama A Alves A Schmitt F. Identification of molecular phenotypes in canine mammary carcinomas with clinical implications: application of the human classification. Virchows Arch. (2008) 453:123–32. doi: 10.1007/s00428-008-0644-3, PMID: 18677512

[19] Kim NH Lim HY Im KS Kim JH Sur JH. Identification of triple-negative and basal-like canine mammary carcinomas using four basal markers. J Comp Pathol. (2013) 148:298–306. doi: 10.1016/j.jcpa.2012.08.009, PMID: 23079102

[20] Sassi F Benazzi C Castellani G Sarli G. Molecular-based tumour subtypes of canine mammary carcinomas assessed by immunohistochemistry. BMC Vet Res. (2010) 6:5. doi: 10.1186/1746-6148-6-5 20109214 PMC2837647

[21] Zhang H Pei S Zhou B Wang H Du H Zhang D. Establishment and characterization of a new triple-negative canine mammary cancer cell line. Tissue Cell. (2018) 54:10–9. doi: 10.1016/j.tice.2018.07.003, PMID: 30309498

[22] Mucha J Rybicka A Dolka I Szymańska J Manuali E Parzeniecka-Jaworska M. Immunosuppression in dogs during mammary cancer development. Vet Pathol. (2016) 53:1147–53. doi: 10.1177/0300985816634808, PMID: 27106740

[23] Giambrone G Di Giorgio S Vullo C Marino G Puleio R Mariotti F. Does TLS exist in canine mammary gland tumours? Preliminary results in simple carcinomas. Vet. Sci. (2022) 9:628. doi: 10.3390/vetsci9110628, PMID: 36423077 PMC9697810

[24] Muscatello LV Avallone G Brunetti B Bacci B Foschini MP Sarli G. Standardized approach for evaluating tumor infiltrating lymphocytes in canine mammary carcinoma: spatial distribution and score as relevant features of tumor malignancy. Vet J. (2022) 283-284:105833. doi: 10.1016/j.tvjl.2022.105833 35489672

[25] Schmid P Adams S Rugo HS Schneeweiss A Barrios CH Iwata H. Atezolizumab and nab-paclitaxel in advanced triple-negative breast cancer. N Engl J Med. (2018) 379:2108–21. doi: 10.1056/NEJMoa1809615, PMID: 30345906

[26] Zimmerli D Brambillasca CS Talens F Bhin J Linstra R Romanens L. MYC promotes immune-suppression in triple-negative breast cancer via inhibition of interferon signaling. Nat Commun. (2022) 13:6579. doi: 10.1038/s41467-022-34000-6 36323660 PMC9630413

[27] Franzoni MS Brandi A De Oliveira Matos Prado JK Elias F Dalmolin F De Faria Lainetti P. Tumor-infiltrating CD4+ and CD8+ lymphocytes and macrophages are associated with prognostic factors in triple-negative canine mammary complex type carcinoma. Res Vet Sci. (2019) 126:29–36. doi: 10.1016/j.rvsc.2019.08.021, PMID: 31425936

[28] Kwon JY Moskwa N Kang W Fan TM Lee C. Canine as a comparative and translational model for human mammary tumor. J Breast Cancer. (2023) 26:1–13. doi: 10.4048/jbc.2023.26.e4, PMID: 36762784 PMC9981990

[29] Tran CM Moore AS Frimberger AE. Surgical treatment of mammary carcinomas in dogs with or without postoperative chemotherapy. Vet Comp Oncol. (2016) 14:252–62. doi: 10.1111/vco.12092, PMID: 24735412

[30] Thomas R Al-Khadairi G Decock J. Immune checkpoint inhibitors in triple negative breast cancer treatment: promising future prospects. Front Oncol. (2020) 10:600573. doi: 10.3389/fonc.2020.600573, PMID: 33718107 PMC7947906

[31] Rahdari M Sadat Hashemi H Hashemi SMA Nadjafi-Semnani A Jamalie S Sakhaee MH. Advancements in the utilization of metal nanoparticles for breast cancer treatment: an in vivo studies update. J Lab Anim Res. (2023) 2:63–71. doi: 10.58803/jlar.v2i5.31

[32] Im KS Kim NH Lim HY Kim HW Shin JI Sur JH. Analysis of a new histological and molecular-based classification of canine mammary neoplasia. Vet Pathol. (2014) 51:549–59. doi: 10.1177/0300985813498780, PMID: 24003019

[33] Ammons DT Hopkins LS Cronise KE Kurihara J Regan DP Dow S. Single-cell RNA sequencing reveals the cellular and molecular heterogeneity of treatment-naïve primary osteosarcoma in dogs. Commun Biol. (2024) 7:496. doi: 10.1038/s42003-024-06182-w 38658617 PMC11043452

[34] Ammons DT Harris RA Hopkins LS Kurihara J Weishaar K Dow S. A single-cell RNA sequencing atlas of circulating leukocytes from healthy and osteosarcoma affected dogs. Front Immunol. (2023) 14:1162700. doi: 10.3389/fimmu.2023.1162700 37275879 PMC10235626

[35] Manchester AC Ammons DT Lappin MR Dow S. Single cell transcriptomic analysis of the canine duodenum in chronic inflammatory enteropathy and health. Front Immunol. (2024) 15:1397590. doi: 10.3389/fimmu.2024.1397590, PMID: 38933260 PMC11199541

[36] Miguelena Chamorro B Hameed SA Dechelette M Claude J-B Piney L Chapat L. Characterization of canine Peyer’s patches by multidimensional analysis: insights from immunofluorescence, flow cytometry, and single-cell RNA sequencing. Immunohorizons. (2023) 7:788–805. doi: 10.4049/immunohorizons.2300091, PMID: 38015460 PMC10696420

[37] Zhou Q-J Liu X Zhang L Wang R Yin T Li X. A single-nucleus transcriptomic atlas of the dog hippocampus reveals the potential relationship between specific cell types and domestication. Natl Sci Rev. (2022) 9:nwac147. doi: 10.1093/nsr/nwac147 36569494 PMC9772819

[38] Cambien B Lebrigand K Baeri A Nottet N Compin C Lamit A. Identification of oncolytic vaccinia restriction factors in canine high-grade mammary tumor cells using single-cell transcriptomics. PLoS Pathog. (2020) 16:e1008660. doi: 10.1371/journal.ppat.1008660, PMID: 33075093 PMC7595618

[39] Früh SP Saikia M Eule J Mazulis CA Miller JE Cowulich JM. Elevated circulating Th2 but not group 2 innate lymphoid cell responses characterize canine atopic dermatitis. Vet Immunol Immunopathol. (2020) 221:110015. doi: 10.1016/j.vetimm.2020.110015, PMID: 32058160

[40] Eschke M Moore PF Chang H Alber G Keller SM. Canine peripheral blood TCRαβ T cell atlas: identification of diverse subsets including CD8A+ MAIT-like cells by combined single-cell transcriptome and V(D)J repertoire analysis. Front Immunol. (2023) 14:1123366. doi: 10.3389/fimmu.2023.1123366 36911660 PMC9995359

[41] Chen D Sun J Zhu J Ding X Lan T Wang X. Single cell atlas for 11 non-model mammals, reptiles and birds. Nat Commun. (2021) 12:7083. doi: 10.1038/s41467-021-27162-2 34873160 PMC8648889

[42] Fastrès A Pirottin D Fievez L Marichal T Desmet CJ Bureau F. Characterization of the bronchoalveolar lavage fluid by single cell gene expression analysis in healthy dogs: a promising technique. Front Immunol. (2020) 11:1707. doi: 10.3389/fimmu.2020.01707, PMID: 32849601 PMC7406785

[43] Fastrès A Pirottin D Fievez L Tutunaru A-C Bolen G Merveille A-C. Identification of pro-fibrotic macrophage populations by single-cell transcriptomic analysis in West Highland white terriers affected with canine idiopathic pulmonary fibrosis. Front Immunol. (2020) 11:611749. doi: 10.3389/fimmu.2020.611749, PMID: 33384697 PMC7770158

[44] Hao Y Stuart T Kowalski MH Choudhary S Hoffman P Hartman A. Dictionary learning for integrative, multimodal and scalable single-cell analysis. Nat Biotechnol. (2024) 42:293–304. doi: 10.1038/s41587-023-01767-y, PMID: 37231261 PMC10928517

[45] Germain P-L Lun A Garcia Meixide C Macnair W Robinson MD. Doublet identification in single-cell sequencing data using scDblFinder. F1000Res. (2021) 10:979. doi: 10.12688/f1000research.73600.1, PMID: 35814628 PMC9204188

[46] Aran D Looney AP Liu L Wu E Fong V Hsu A. Reference-based analysis of lung single-cell sequencing reveals a transitional profibrotic macrophage. Nat Immunol. (2019) 20:163–72. doi: 10.1038/s41590-018-0276-y, PMID: 30643263 PMC6340744

[47] Borcherding N Vishwakarma A Voigt AP Bellizzi A Kaplan J Nepple K. Mapping the immune environment in clear cell renal carcinoma by single-cell genomics. Commun Biol. (2021) 4:122. doi: 10.1038/s42003-020-01625-6 33504936 PMC7840906

[48] Liberzon A Birger C Thorvaldsdóttir H Ghandi M Mesirov JP Tamayo P. The molecular signatures database (MSigDB) hallmark gene set collection. Cell Syst. (2015) 1:417–25. doi: 10.1016/j.cels.2015.12.004, PMID: 26771021 PMC4707969

[49] Sheet S Krishnamoorthy S Cha J Choi S Choi B-H. Identification of candidate genes and pathways associated with obesity-related traits in canines via gene-set enrichment and pathway-based GWAS analysis. Animals. (2020) 10:71. doi: 10.3390/ani10112071, PMID: 33182249 PMC7695335

[50] Lee K-H Park H-M Son K-H Shin T-J Cho J-Y. Transcriptome signatures of canine mammary gland tumors and its comparison to human breast cancers. Cancers. (2018) 10:317. doi: 10.3390/cancers10090317, PMID: 30205506 PMC6162473

[51] Tawa GJ Braisted J Gerhold D Grewal G Mazcko C Breen M. Transcriptomic profiling in canines and humans reveals cancer specific gene modules and biological mechanisms common to both species. PLoS Comput Biol. (2021) 17:e1009450. doi: 10.1371/journal.pcbi.1009450, PMID: 34570764 PMC8523068

[52] Graim K Gorenshteyn D Robinson DG Carriero NJ Cahill JA Chakrabarti R. Modeling molecular development of breast cancer in canine mammary tumors. Genome Res. (2020) 31:337–47. doi: 10.1101/gr.256388.119 33361113 PMC7849403

[53] Ge SX Jung D Yao R. ShinyGO: a graphical gene-set enrichment tool for animals and plants. Bioinformatics. (2020) 36:2628–9. doi: 10.1093/bioinformatics/btz931, PMID: 31882993 PMC7178415

[54] Bunis DG Andrews J Fragiadakis GK Burt TD Sirota M. dittoSeq: universal user-friendly single-cell and bulk RNA sequencing visualization toolkit. Bioinformatics. (2021) 36:5535–6. doi: 10.1093/bioinformatics/btaa1011, PMID: 33313640 PMC8016464

[55] Jin S Guerrero-Juarez CF Zhang L Chang I Ramos R Kuan C-H. Inference and analysis of cell-cell communication using CellChat. Nat Commun. (2021) 12:1088. doi: 10.1038/s41467-021-21246-9, PMID: 33597522 PMC7889871

[56] Kim M-C de U Borcherding N Wang L Paek J Bhattacharyya I. Single-cell transcriptomics unveil profiles and interplay of immune subsets in rare autoimmune childhood Sjögren’s disease. Commun Biol. (2024) 7:481. doi: 10.1038/s42003-024-06124-6, PMID: 38641668 PMC11031574

[57] Hewitt RJ Lloyd CM. Regulation of immune responses by the airway epithelial cell landscape. Nat Rev Immunol. (2021) 21:347–62. doi: 10.1038/s41577-020-00477-9, PMID: 33442032 PMC7804588

[58] Nguyen TT Shin DH Sohoni S Singh SK Rivera-Molina Y Jiang H. Reshaping the tumor microenvironment with oncolytic viruses, positive regulation of the immune synapse, and blockade of the immunosuppressive oncometabolic circuitry. J Immunother Cancer. (2022) 10:e004935. doi: 10.1136/jitc-2022-004935, PMID: 35902132 PMC9341188

[59] Mohammed SI Utturkar S Lee M Yang HH Cui Z Atallah Lanman N. Ductal carcinoma in situ progression in dog model of breast cancer. Cancers. (2020) 12:418. doi: 10.3390/cancers12020418, PMID: 32053966 PMC7072653

[60] Beetch M Harandi-Zadeh S Yang T Boycott C Chen Y Stefanska B. DNA methylation landscape of triple-negative ductal carcinoma in situ (DCIS) progressing to the invasive stage in canine breast cancer. Sci Rep. (2020) 10:2415. doi: 10.1038/s41598-020-59260-4, PMID: 32051475 PMC7015930

[61] Zanin R Pegoraro S Ros G Ciani Y Piazza S Bossi F. HMGA1 promotes breast cancer angiogenesis supporting the stability, nuclear localization and transcriptional activity of FOXM1. J Exp Clin Cancer Res. (2019) 38:313. doi: 10.1186/s13046-019-1307-8, PMID: 31311575 PMC6636010

[62] Chantada-Vázquez MDP Castro López A García-Vence M Acea-Nebril B Bravo SB Núñez C. Protein Corona gold nanoparticles fingerprinting reveals a profile of blood coagulation proteins in the serum of HER2-overexpressing breast cancer patients. Int J Mol Sci. (2020) 21:449. doi: 10.3390/ijms21228449, PMID: 33182810 PMC7696934

[63] Chang X Liu J Yang Q Gao Y Ding X Zhao J. Targeting HMGA1 contributes to immunotherapy in aggressive breast cancer while suppressing EMT. Biochem Pharmacol. (2023) 212:115582. doi: 10.1016/j.bcp.2023.115582, PMID: 37146833

[64] Merikhian P Eisavand MR Farahmand L. Triple-negative breast cancer: understanding Wnt signaling in drug resistance. Cancer Cell Int. (2021) 21:419. doi: 10.1186/s12935-021-02107-3, PMID: 34376211 PMC8353874

[65] Shurin MR. Osteopontin controls immunosuppression in the tumor microenvironment. J Clin Invest. (2018) 128:5209–12. doi: 10.1172/JCI124918, PMID: 30395537 PMC6264653

[66] Amirkhani Namagerdi A d’Angelo D Ciani F Iannuzzi CA Napolitano F Avallone L. Triple-negative breast cancer comparison with canine mammary Tumors from light microscopy to molecular pathology. Front Oncol. (2020) 10:563779. doi: 10.3389/fonc.2020.563779, PMID: 33282730 PMC7689249

[67] Carvalho MI Pires I Prada J Gregório H Lobo L Queiroga FL. Intratumoral FoxP3 expression is associated with angiogenesis and prognosis in malignant canine mammary tumors. Vet Immunol Immunopathol. (2016) 178:1–9. doi: 10.1016/j.vetimm.2016.06.006, PMID: 27496736

[68] Raposo TP Pires I Carvalho MI Prada J Argyle DJ Queiroga FL. Tumour-associated macrophages are associated with vascular endothelial growth factor expression in canine mammary tumours. Vet Comp Oncol. (2015) 13:464–74. doi: 10.1111/vco.12067, PMID: 24119241

[69] Christianson J Oxford JT Jorcyk CL. Emerging perspectives on leukemia inhibitory factor and its receptor in cancer. Front Oncol. (2021) 11:693724. doi: 10.3389/fonc.2021.693724, PMID: 34395259 PMC8358831

[70] Somasundaram V Ridnour LA Cheng RY Walke AJ Kedei N Bhattacharyya DD. Systemic Nos2 depletion and cox inhibition limits TNBC disease progression and alters lymphoid cell spatial orientation and density. Redox Biol. (2022) 58:102529. doi: 10.1016/j.redox.2022.102529, PMID: 36375380 PMC9661390

[71] Meng J Jiang Y-Z Zhao S Tao Y Zhang T Wang X. Tumor-derived Jagged1 promotes cancer progression through immune evasion. Cell Rep. (2022) 38:110492. doi: 10.1016/j.celrep.2022.110492, PMID: 35263601

[72] Klement JD Paschall AV Redd PS Ibrahim ML Lu C Yang D. An osteopontin/CD44 immune checkpoint controls CD8+ T cell activation and tumor immune evasion. J Clin Invest. (2018) 128:5549–60. doi: 10.1172/JCI123360, PMID: 30395540 PMC6264631

[73] Weber GF Ashkar S Glimcher MJ Cantor H. Receptor-ligand interaction between CD44 and osteopontin (Eta-1). Science. (1996) 271:509–12. doi: 10.1126/science.271.5248.509, PMID: 8560266

[74] Weimann TK Wagner C Goos M Wagner SN. CD44 variant isoform v10 is expressed on tumor-infiltrating lymphocytes and mediates hyaluronan-independent heterotypic cell-cell adhesion to melanoma cells. Exp Dermatol. (2003) 12:204–12. doi: 10.1034/j.1600-0625.2003.00044.x, PMID: 12702150

[75] Zhang X-X Luo J-H Wu L-Q. FN1 overexpression is correlated with unfavorable prognosis and immune infiltrates in breast cancer. Front Genet. (2022) 13:913659. doi: 10.3389/fgene.2022.1103783, PMID: 36035176 PMC9417469

[76] Hussain S Saxena S Shrivastava S Mohanty AK Kumar S Singh RJ. Gene expression profiling of spontaneously occurring canine mammary tumours: insight into gene networks and pathways linked to cancer pathogenesis. PLoS One. (2018) 13:e0208656. doi: 10.1371/journal.pone.0208656 30517191 PMC6281268

[77] Monteiro LN Salgado BS Oliveira DE Rivera-Calderon LG Montoya-Flórez LM Sanctis P. Osteopontin expression and its relationship with prognostic biomarkers in canine mammary carcinomas. Pesq Vet Bras. (2020) 40:210–9. doi: 10.1590/1678-5150-PVB-6489

[78] Song Q Ruiz J Xing F Lo H-W Craddock L Pullikuth AK. Single-cell sequencing reveals the landscape of the human brain metastatic microenvironment. Commun Biol. (2023) 6:760. doi: 10.1038/s42003-023-05124-2 37479733 PMC10362065

[79] Zhong Y Li F Zhang S Yang Z Ren X Cao X. Syndecan-1 as an immunogene in triple-negative breast cancer: regulation tumor-infiltrating lymphocyte in the tumor microenviroment and EMT by TGFb1/Smad pathway. Cancer Cell Int. (2023) 23:76. doi: 10.1186/s12935-023-02917-7, PMID: 37069585 PMC10111802

[80] Wang C Wallerman O Arendt M-L Sundström E Karlsson Å Nordin J. A novel canine reference genome resolves genomic architecture and uncovers transcript complexity. Commun Biol. (2021) 4:185. doi: 10.1038/s42003-021-01698-x, PMID: 33568770 PMC7875987

