# Supplementary figures and images for: Leveraging single-cell transcriptomic data to uncover immune suppressive cancer cell subsets in triple-negative canine breast cancers
Source: Front Vet Sci. 2024 Sep 23;11:1434617. doi: 10.3389/fvets.2024.1434617 (PMC11457229; doi:10.3389/fvets.2024.1434617)

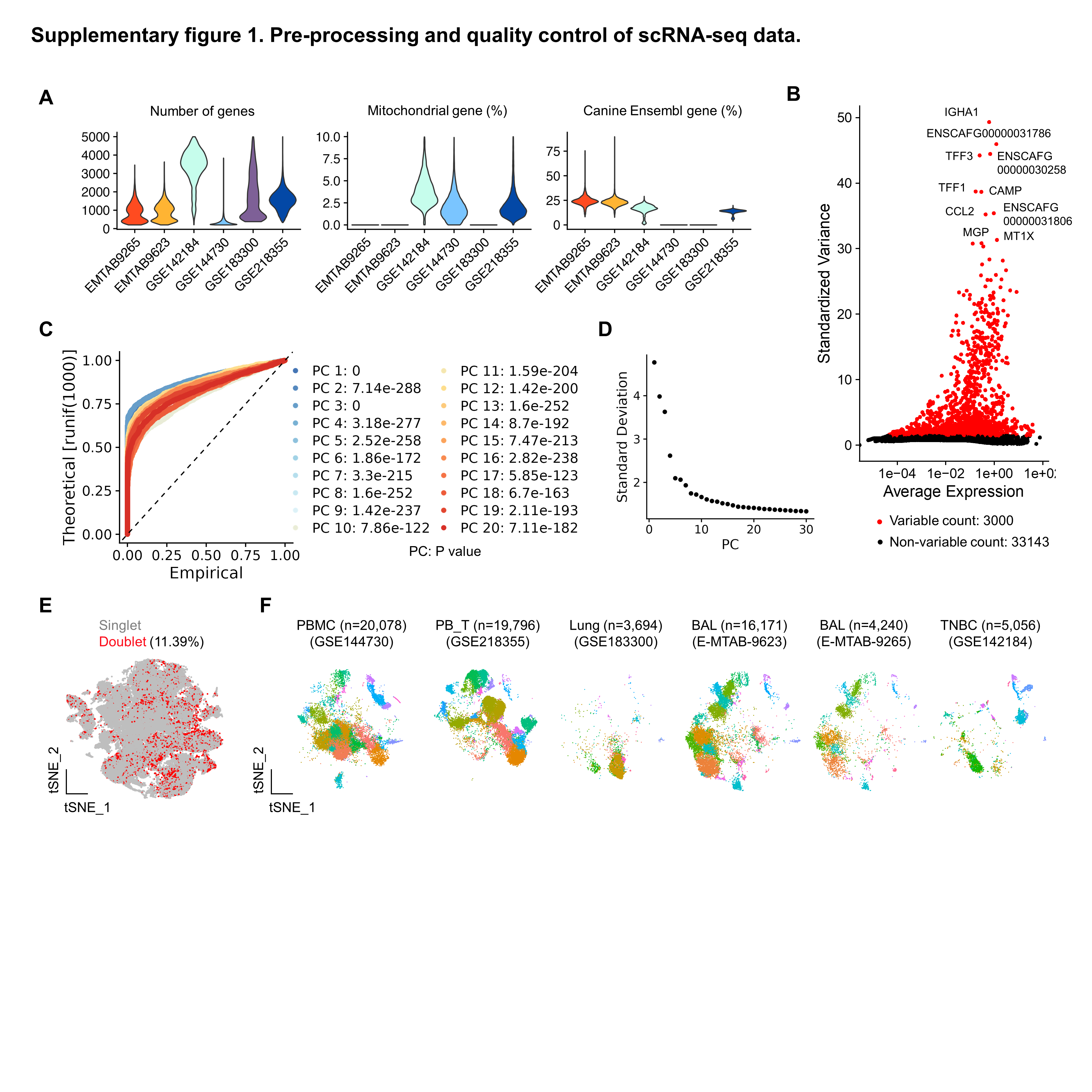

Supplement: Supplementary file 5 [file Image_1.TIF]

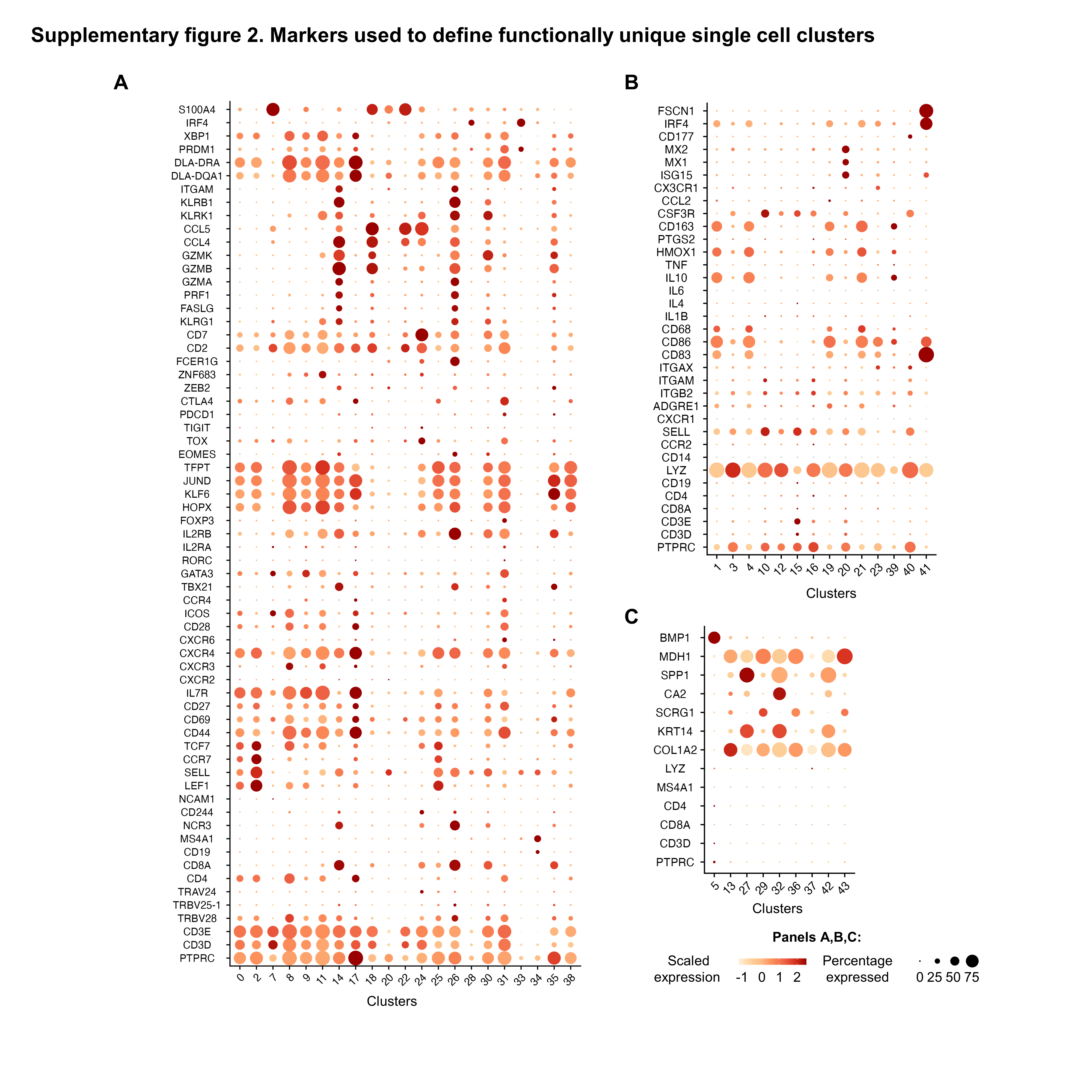

Supplement: Supplementary file 6 [file Image_2.TIF]

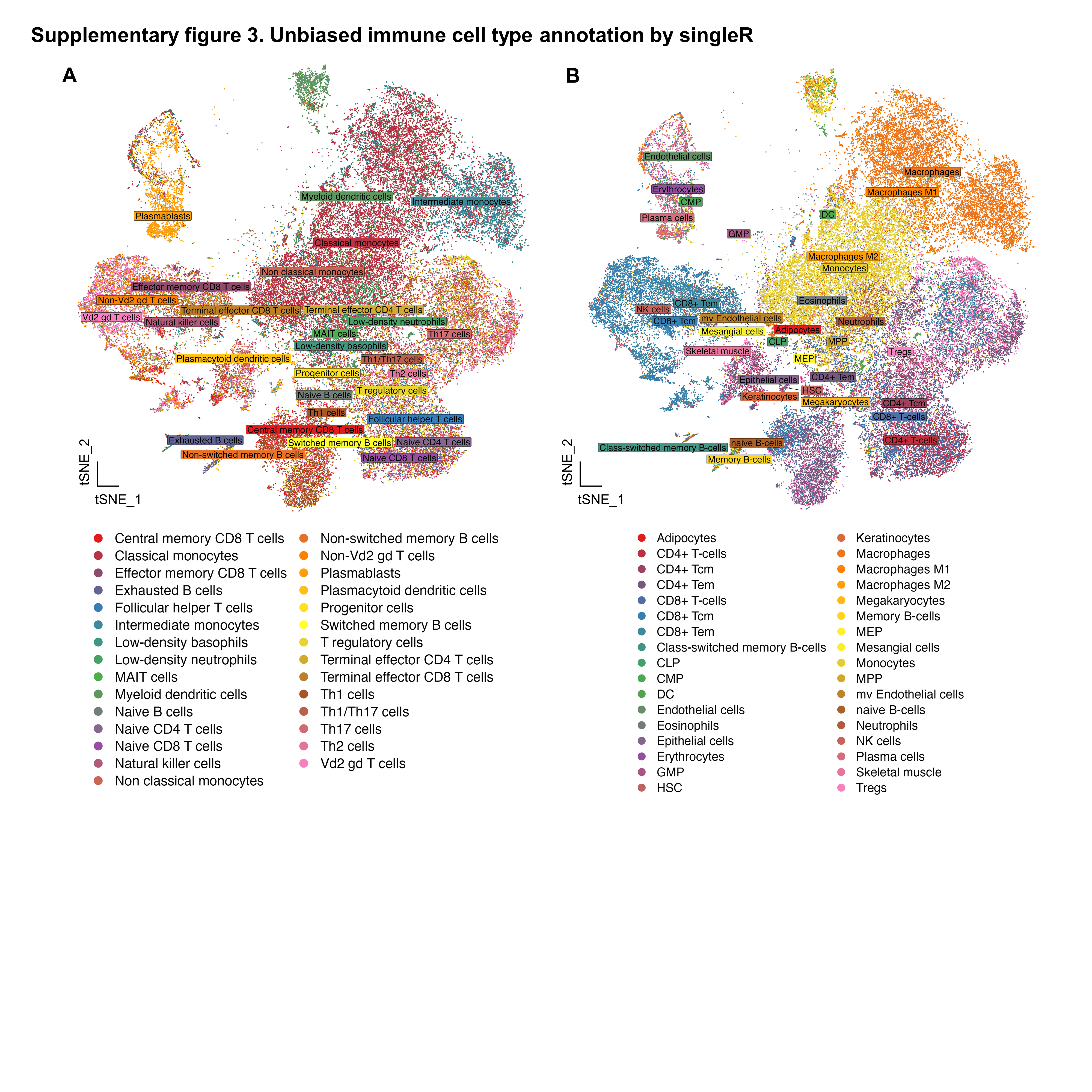

Supplement: Supplementary file 7 [file Image_3.TIF]

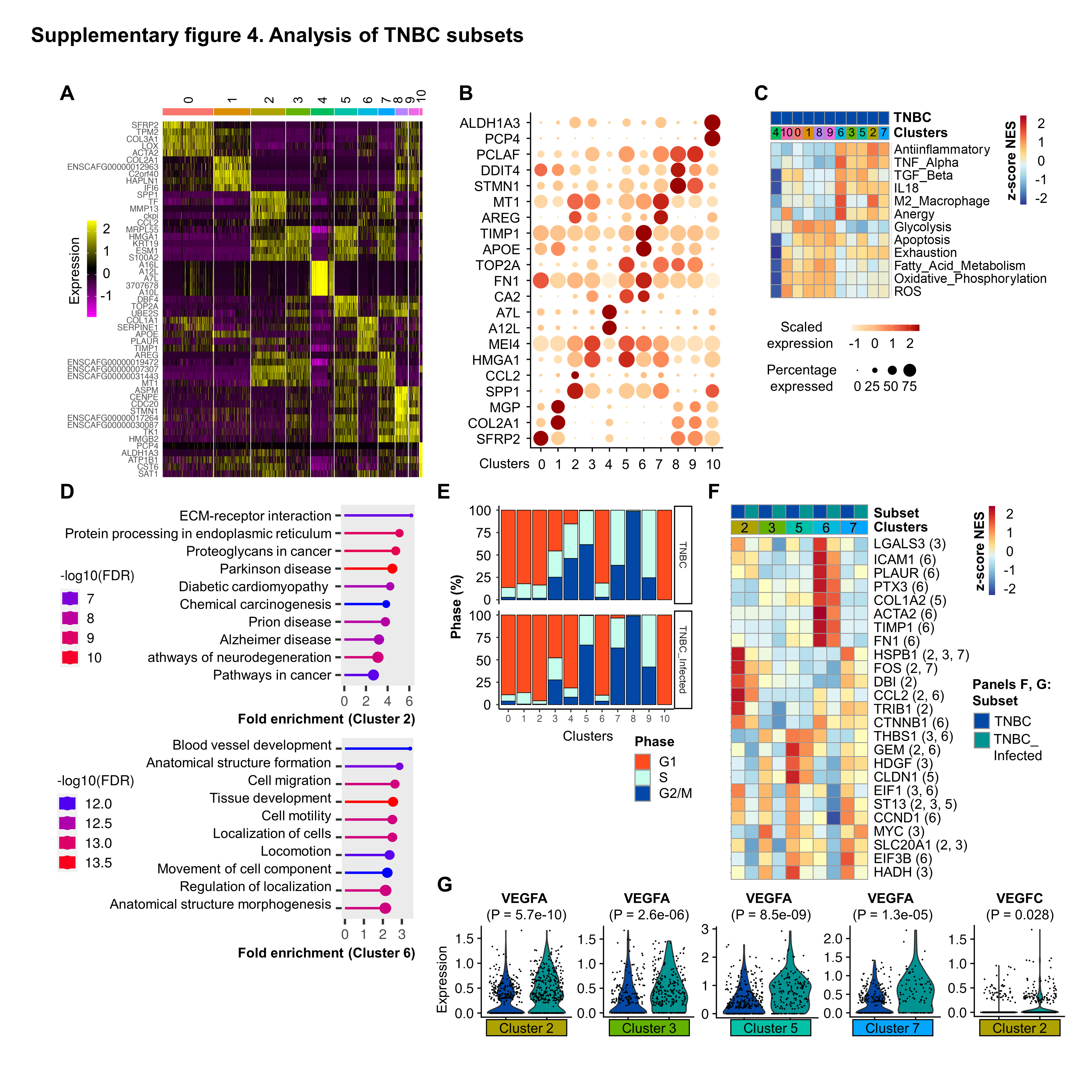

Supplement: Supplementary file 8 [file Image_4.TIF]

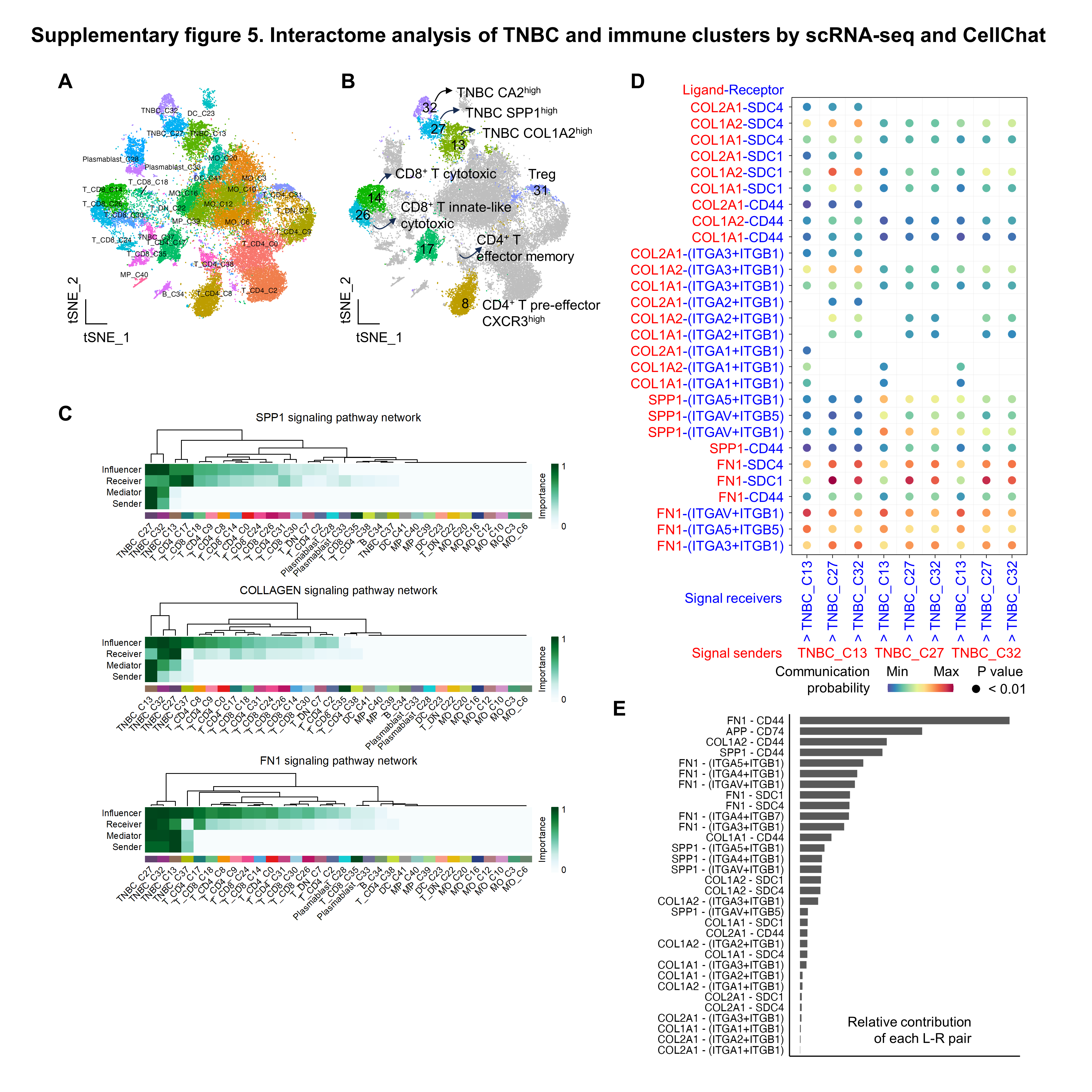

Supplement: Supplementary file 9 [file Image_5.TIF]
